# The impact factors of social media users' forwarding behavior of COVID-19 vaccine topic: Based on empirical analysis of Chinese Weibo users

**DOI:** 10.3389/fpubh.2022.871722

**Published:** 2022-09-14

**Authors:** Kun Sun, Han Wang, Jinsheng Zhang

**Affiliations:** School of Journalism and Communication, Jinan University, Guangzhou, China

**Keywords:** COVID-19 vaccine, social media, emotion, social network structure, forwarding behavior

## Abstract

**Introduction:**

Social media, an essential source of public access to information regarding the COVID-19 vaccines, has a significant effect on the transmission of information regarding the COVID-19 vaccines and helps the public gain correct insights into the effectiveness and safety of the COVID-19 vaccines. The forwarding behavior of social media users on posts concerned with COVID-19 vaccine topics can rapidly disseminate vaccine information in a short period, which has a significant effect on transmission and helps the public access relevant information. However, the factors of social media users' forwarding posts are still uncertain thus far. In this paper, we investigated the factors of the forwarding COVID-19 vaccines Weibo posts on Chinese social media and verified the correlation between social network characteristics, Weibo textual sentiment characteristics, and post forwarding.

**Methods:**

This paper used data mining, machine learning, sentiment analysis, social network analysis, and regression analysis. Using “新冠疫苗 (COVID-19 vaccine)” as the keyword, we used data mining to crawl 121,834 Weibo posts on Sina Weibo from 1 January 2021 to 31 May 2021. Weibo posts not closely correlated with the topic of the COVID-19 vaccines were filtered out using machine learning. In the end, 3,158 posts were used for data analysis. The proportions of positive sentiment and negative sentiment in the textual of Weibo posts were calculated through sentiment analysis. On that basis, the sentiment characteristics of Weibo posts were determined. The social network characteristics of information transmission on the COVID-19 vaccine topic were determined through social network analysis. The correlation between social network characteristics, sentiment characteristics of the text, and the forwarding volume of posts was verified through regression analysis.

**Results:**

The results suggest that there was a significant positive correlation between the degree of posting users in the social network structure and the amount of forwarding. The relationship between the closeness centrality and the forwarding volume was significantly positive. The betweenness centrality was significantly positively correlated with the forwarding volume. There was no significant relationship between the number of posts containing more positive sentiments and the forwarding volume of posts. There was a significant positive correlation between the number of Weibo posts containing more negative sentiments and the forwarding volume.

**Conclusion:**

According to the characteristics of users, COVID-19 vaccine posts from opinion leaders, “gatekeepers,” and users with high-closeness centrality are more likely to be reposted. Users with these characteristics should be valued for their important role in disseminating information about COVID-19 vaccines. In addition, the sentiment contained in the Weibo post is an important factor influencing the public to forward vaccine posts. Special attention should be paid to the negative sentimental tendency contained in this post on Weibo to mitigate the negative impact of the information epidemic and improve the transmission effect of COVID-19 vaccine information.

## Introduction

The global pandemic of COVID-19 has been one of the most hazardous public health emergencies over the past few years, arising from its negative effect on all human health. As of 5 January 2022, COVID-19 has caused nearly 290 million infections and over 5 million deaths worldwide (1). To control the negative effect of the global pandemic of COVID-19, research on vaccines to establish the body's immune system has been found as one of the critical methods to fight against the pandemic (2). Vaccines, one of the most effective and safest preventive measures to prevent and control disease in humans, have been reported to significantly decrease the number of infections transmitted by vaccine-preventable diseases (3). Various vaccines have been developed, tested, and endorsed in numerous nations after the epidemic outbreak. Furthermore, a mass vaccination campaign against COVID-19 has been rapidly launched worldwide.

China has begun to develop the COVID-19 vaccines at a very early stage after the epidemic outbreak. On 31 December 2020, as announced by the Information Office of the State Council of the People's Republic of China, the national vaccines of COVID-19 were approved by the National Medical Products Administration for conditional marketing (4). To control the COVID-19 pandemic, herd immunization for COVID-19 requires a large proportion of the population to be vaccinated (5). However, numerous factors can practically affect the outcome of vaccination programs promoted by official agencies (6). Studies taken during the vaccine development about the willingness of the public on COVID-19 vaccination showed different attitudes among different countries, and the people who were willing to take the vaccines ranged between 41 and 89% (7). Accordingly, despite the remarkable preventive and controlling effects of vaccines on the transmission of diseases, a part of the public remains reluctant to have the COVID-19 vaccination and is skeptical about vaccination. This phenomenon is termed “vaccine hesitancy” (8). As reported by existing studies, individuals with different levels of hesitancy to receive specific vaccines may generally cause lower immunization rates and slow uptake of newly introduced vaccines (9–12). The reasons for the increase in vaccine hesitancy have been studied, which include the lack of knowledge about vaccines (13) and the difficulty of accessing information online (14). Thus, effective transmission of knowledge regarding COVID-19 vaccines and promoting public understanding of COVID-19 vaccines to increase vaccination rates are vital topics worth discussing.

There is a wealth of health and medical information on social media (15), and it turns out to be one of the most popular places to access health information (16). Some studies reported that health information acquired from social media platforms might exacerbate people's hesitancy (17) about vaccines, but this conclusion needs to be confirmed in the context of COVID-19 vaccination. Undoubtedly, social media can rapidly disseminate considerable information regarding vaccine issues, and it is an effective tool for public health departments to promote public health awareness by sending brief messages (18). In the internet age, social media can spread much information regarding vaccine topics in a short time, which is directly correlated with the communicative action of users. Communicative action is the interaction between two linguistically and behaviorally competent subjects to achieve mutual understanding and coherence (19). For instance, Sina Weibo (China's most popular social media platform, similar to Twitter) acts as a platform for sharing and acquiring information based on user relationships. Weibo users interact by following, posting, forwarding, commenting, and liking. They communicate in cyberspace and develop connections among themselves, spreading information widely. Transmission behavior adequately explains individual users' participation in information transmission in cyberspace, which accounts for people's involvement in information transmission by analyzing the six behavioral elements, that is, “contacting, sharing, participating, searching, protecting, and forwarding.” Among the six behavioral elements above, forwarding behavior acts as a simple and effective behavior, which is important to study the inner mechanism of information transmission.

Forwarding behavior indicates that the forwarding user (user b) is interested in the specific message posted by the original promulgator (user a) (20). As indicated by existing research, the number of forwards accounts for 35% of the number of posts on Twitter, while this percentage reaches up to 65% in Sina Weibo (21). In the online health information sphere, the transmission of information is more significantly dependent on information forwarding because of the specificity of health information (22). Furthermore, from the perspective of information processing, users' forwarding behavior is of high research significance. Forwarding has been found as a comprehensive behavior of information processing involving reading and publishing information in the process of forwarding. When users decide which post to forward, they need to pay more attention to the content of the post and the authority of the source (23). Accordingly, the behavior of forwarding is a deep and active engagement behavior (24). Existing research on cancer topics indicated that people engage in cancer information transmission on social media to meet related information demands and transmission demands (25). Thus, users' forwarding behavior is of positive significance to health information acquisition level.

This study has three main contributions. The first contribution is that this paper used big data mining and analysis to creatively study the reasons why the public reposts COVID-19 vaccine-related posts, thus contributing to knowledge for research on the effect of information transmission. Second, this paper integrated two variables of post content and user characteristics, providing a valuable perspective for us to comprehensively understand people's information transmission behavior in the social media era. Finally, posts on COVID-19 vaccine-related topics objectively reflect the public's concern about COVID-19 vaccines in China, and COVID-19 vaccine information on social media is crucial for increasing scientific awareness of COVID-19 vaccines and effectively increasing vaccination rates. Thus, this paper also offers advice on how to use social media platforms to increase the spread of COVID-19 vaccine posts.

## Literature review

Numerous studies have been conducted to investigate the forwarding behavior of social media users. A primary focus is on finding the rationale for forwarding information on a specific topic. Research on the forwarding behavior of social media users is conducive to revealing the underlying mechanisms of health information transmission. As reported by existing studies, a combination of factors impacts users' forwarding behavior. Specifically, the influencing factors of Weibo posts forwarding can be roughly divided into two categories: user characteristics and information characteristics (26, 27). This paper analyzes the factors of social media users' forwarding of posts about the COVID-19 vaccine topic from social network characteristics and Weibo textual sentiment.

### Network characteristics and forwarding behavior

An overriding factor accounting for the forwarding behavior of social media users is the structure of social networks. In social media, users are not isolated from each other, whereas they are connected by disseminating information, which generates a directed social network diagram of information transmission. In the social network relationship, each node in the network represents a user, and users can establish connections by forwarding posts, thus forming a social network structure with a forwarding relationship. The “position” and power can be measured with three metrics, that is, degree, betweenness centrality, and closeness centrality in the network structure (28).

In the social network structure, the degree in the network structure can be analyzed to investigate the scope of users' social activities. The higher the degree of a user is, the more the user is in the core position of the entire social network structure of information transmission, and the more users directly establish contact with it (29). Thus, the higher the degree indicating that the user is more likely to act as an opinion leader (30). As the COVID-19 vaccine topic has a threshold of expertise for users' information transmission, users are alert to the content of forwarding and more careful in choosing the sources of information. They prefer complying with official institutions or medical experts with professional knowledge (31). Such a type of user can control more information resources and discourse authority. They are the primary sources for ordinary users to obtain news and play an opinion leader in information transmission between the public, medical institutions, and health communities. Accordingly, the higher the degree of the posting user's social network characteristics, the higher the possibility that his posts relating to COVID-19 vaccines are being forwarded.

Betweenness centrality is a way of detecting the amount of influence a node has over the flow of information in a graph. It is often used to find nodes that serve as a bridge from one part of a graph to another (32). In social network structure, betweenness centrality focuses on the shortest path between two nodes and the frequency in information transmission (33). If a member is on multiple shortest paths of other members, it is a core member and has high betweenness centrality. By measuring the centrality above, the effective gatekeepers in a social network can be identified (34). American Psychologist Levin initially proposed the gatekeeper theory (35). Gatekeepers will determine the channels of information flow, as well as what information should be disseminated. Thus, gatekeepers' knowledge, opinions, and attitudes significantly impact information transmission. In addition, users having high betweenness centrality are indicated as the bridge between different groups in the network, maintaining contact with professional health service providers and interacting closely with grassroots opinion leaders (36) (The grassroots is defined as the basic level of society or of an organization). In other words, users having higher betweenness centrality often potentially control and impact the nodes not adjacent to each other in the social network (33). Therefore, it can be speculated that the higher the betweenness centrality of a posting user, the greater the likelihood that a COVID-19 vaccine-related post will be forwarded.

Closeness centrality emphasizes the average distance between one node and the others in the social network structure (37). The closeness centrality of a node measures its average farness (inverse distance) to all other nodes. Nodes with a high-closeness score have the shortest distances to all other nodes (38). In general, the inverse of the average distance to others in the network is used as a measure of closeness centrality. A high-closeness centrality means that a person is directly connected or “just a hop away” from most others in the network. In contrast, vertices in very peripheral locations may have low closeness centrality scores, indicating the high number of hops or connections they need to take to connect to distant others in the network (39). Previous studies have shown that users with high-closeness centrality are relatively independent in the actual complex network structure of information transmission and can obtain useful information through a few nodes (40). Therefore, users with high-closeness centrality have advantages in acquiring information and knowledge, but their advantages in information transmission have not been proven.

### Textual sentiment and forwarding behavior

The cognitive theory of emotion suggests that the body receives information from the external world into the perceptual system. The body will be organized and compiled by the perceptual and mental systems. Besides, the information will lead to the body's positive or negative sentiment response, thus causing some tendencies to behavior (41). The theory above highlights the strong effect of emotions on behavior (42). The type of user posts sentiment in social media represents the user's sentiment tendency in sentiment transmission (43), which is generally classified to be positive, neutral, or negative. The correlation between sentiment and social media users' forwarding behavior has been established, whereas there have been different findings of the correlation. Jonah Berger found positive textual view gets more forwarding volumes than negative sentiment (44), while Ferrara and Yang reported negative sentiment posts to gain more forwarding volumes and faster forwarding than positive sentiment (45). According to Xuemei T and Lili Z (46), the above change of findings might be correlated with subject differences, that is, Jonah Berger's object of study was email forwarding of news. In contrast, Laurie Kramer selected the forwarding of Twitter users' comments as the object. As a result, it can be presumed that the correlation between different sentiment posts and the sum of posts' direct forwarding volume in the social network structure of various topics.

In brief, despite the research conducted in the literature on the correlation between social network structure, textual sentiment, and user-forwarding behavior, this area still has some researchable space. By reviewing existing studies, we indicated that though similar studies have discussed the effects arising from betweenness centrality and closeness centrality on post forwarding (36), the research on the forwarding behavior of COVID-19 vaccine topics on social platforms from the social network structure should be enriched. Although the study on the correlation between sentiment and forwarding behavior has been conducted on political issues (47), natural disasters (48), and hot social news (49), rare studies focused on vaccine issues. This paper combined social network characteristics with textual sentiment to investigate the factors of social media users' forwarding of posts relating to COVID-19 vaccines. This paper mainly aims to investigate two questions as follows:

***RQ1: What is the relationship between the different social network*** ***characteristics of posting users and the forwarding volume of COVID-19*** ***vaccines topic?***
***RQ2: What relationship do different sentiment posts and the sum of posts'*** ***direct forwarding volume in the COVID-19 vaccines topic network structure?***

## Methods

### Sample and data

**Figure 1:**
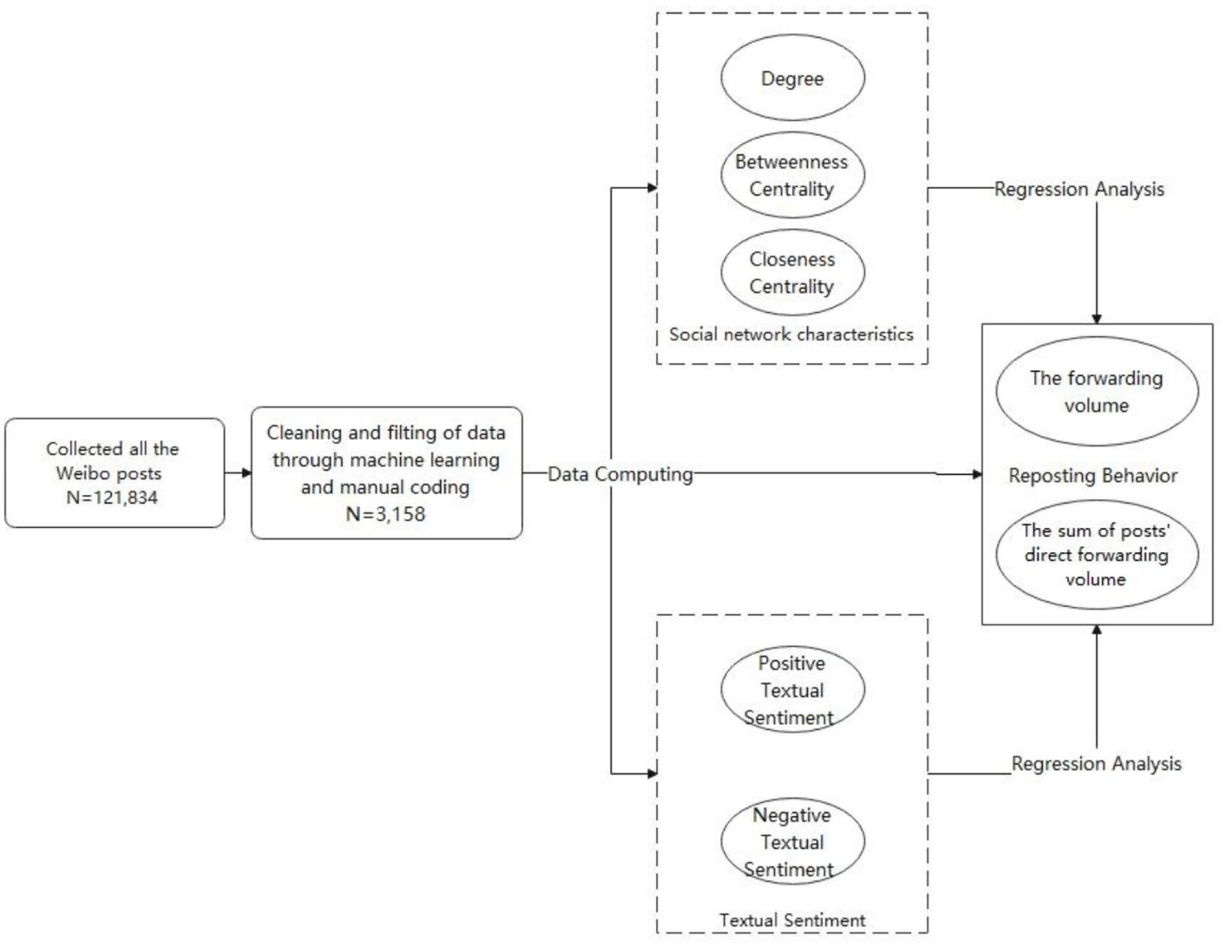
Research design framework.

Since the outbreak of COVID-19 in China, topics related to COVID-19 vaccines have been hotly discussed by the public on social media. In particular, vaccination campaigns across China began on 1 January 2021, when Beijing fully launched the COVID-19 vaccination campaign for nine key groups. The safety, efficacy, and side effects of vaccines have been further debated. Sina Weibo has been the largest social media platform in China with 230 million daily active users (50, 51). On that basis, this paper chooses Sina Weibo as the data acquisition platform and uses the Python language based on regular expression to write the crawler to grab the post text (All Python scripts involved in this paper are written by the built-in integration library of Anaconda3 5.3.0). As time went on, the vaccination campaign gradually expanded, and the number of people vaccinated gradually increased. In the absence of mass adverse reactions, concerns about vaccine safety declined (52). Therefore, this paper selects the topic period as 5 months, that is, from 1 January 2021 to 31 May 2021. This paper covers the public's cognitive process of new affairs in the time range, reflects the change process of public attitude and acceptance emotion, and ensures the representativeness of data samples. This paper collects 121,834 Weibo posts using the Application Programming Interface (API) of Sina Weibo, with the keyword “新冠疫苗 (COVID-19 vaccines)” retrieved in Chinese. Figure 1 shows the overall research design framework of this paper. After the cleaning of the data set and machine learning and other pre-processing work, the sample number of this paper is determined to be 3158 Weibo posts highly relevant to the research topic. The steps of data cleaning and pretreatment are as follows:

#### Data cleaning

The information of Weibo posts captured by us includes user name, user id, number of fans, post time, post content, post id, forwarding volume of posts, number of likes, and number of comments. Through data cleaning, we deleted Weibo posts with missing user names, repetitive posts, meaningless posts containing only pure emoticons, and posts with zero forwarding volume. A total of 13002 posts were left. To make the data samples more consistent with the requirements of the research topic, we used logistic regression of machine learning and random forest model algorithm combined with manual verification to preprocess the remaining 13002 posts.

#### Content classification

We divided the data after cleaning into two groups by content: One group is the content-related group which was highly related to the posting content and the research topic; the other group is the content irrelevant group which was unrelated to the research topic. Referring to the study of SAGE Working Group (8), we defined Weibo posts about vaccine safety, effectiveness, side effects, trust, vaccination willingness, and vaccination sentiment as research samples are highly related to the research topic. Posts that do not involve the above contents are defined as irrelevant to the research topic and are not included in the scope of this paper.

#### Determine training samples for machine learning

Through the method of manual coding, it can provide effective training samples for machine learning. Therefore, we selected 3000 posts from 13002 posts by random sampling method. Three coders familiar with content classification standards were invited to encode the content of 3,000 extracted posts. The coding rule is as follows: If the content of the post is related to the research topic, the code is “1”; if the content of the post is not related to the research topic, the code is “0.” When counting the classification result of the content of a post, the coders' vote of at least two votes (≥2) will be counted as the final classification result of the post. After the coding, we randomly selected 10% of the posts whose coding results had been determined and invited another independent coder to test them. According to Holsti formula (53), the internal reliability of coders was 95%, which is reliable and can be used as training samples for machine learning. To ensure the balance of machine learning sample data categories, we randomly selected 500 posts from the content-related group and 500 posts from the content irrelevant group. These 1,000 posts constitute the final training sample for machine learning.

#### Test of the effect of the machine learning model

**Figure 2:**
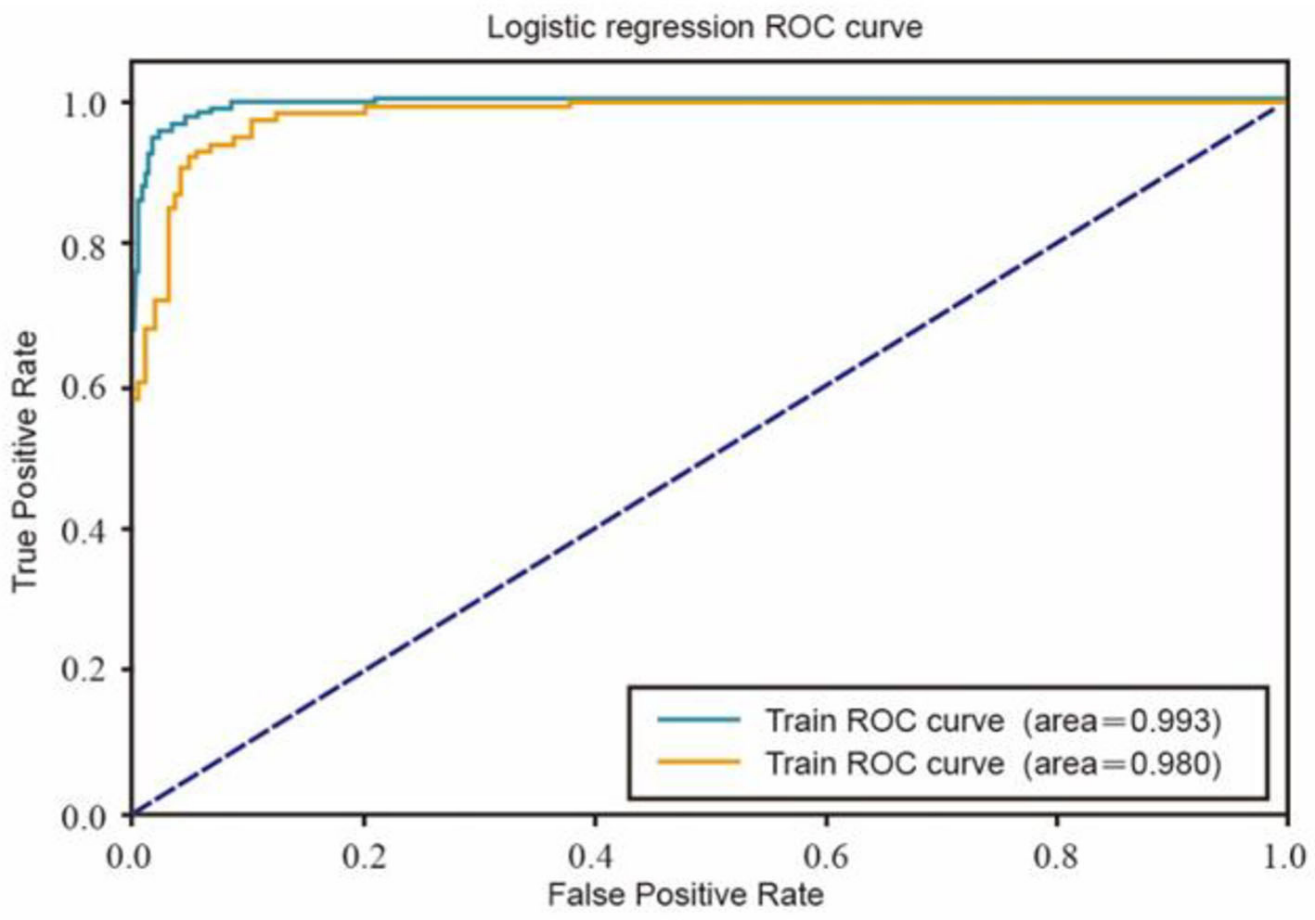
Logistic regression ROC curve.

**Figure 3:**
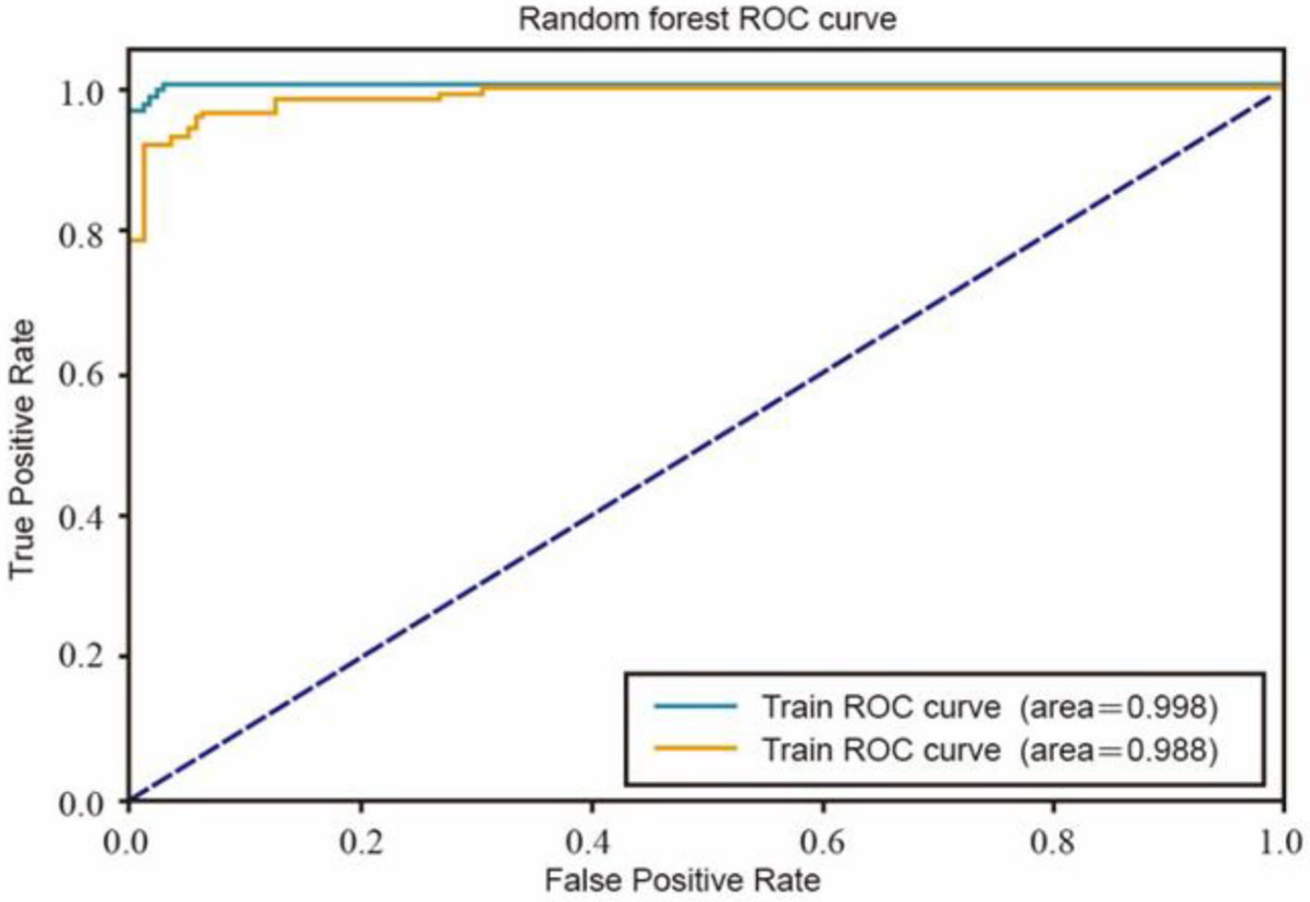
Random forest ROC curve.

The determined 1000 posts were put into the machine learning algorithm as training samples. The logistic regression ROC curve (Figure 2) and random forest ROC curve (Figure 3) obtained were both close to the upper left corner, and the ACU values (area under ROC Curve) were > 0.9. These results indicate that the machine learning model is effective and can be used to classify the correlation of the content of the whole sample by machine learning.

#### Determination of the study sample

After verifying the availability of the machine learning model, we classified the content of 13002 posts by subject-dependent machine learning. The final results of classification were 3344 posts highly related to the research topic, and 9658 posts irrelevant to the research topic (some posts in the classification result are shown in the Supplementary material). For the classification results, we invited three coders again to manually check and discuss the results. The consistent conclusion is that the classification result of machine learning is reasonable and has strong usability. However, to better measure the influence of different variables on forwarding behavior, self-forwarding of posting users should be excluded from 3344 posts related to the subject content. Therefore, 3158 posts were left as the final samples.

### Measures of variables

#### Dependent variable

**Figure 4:**
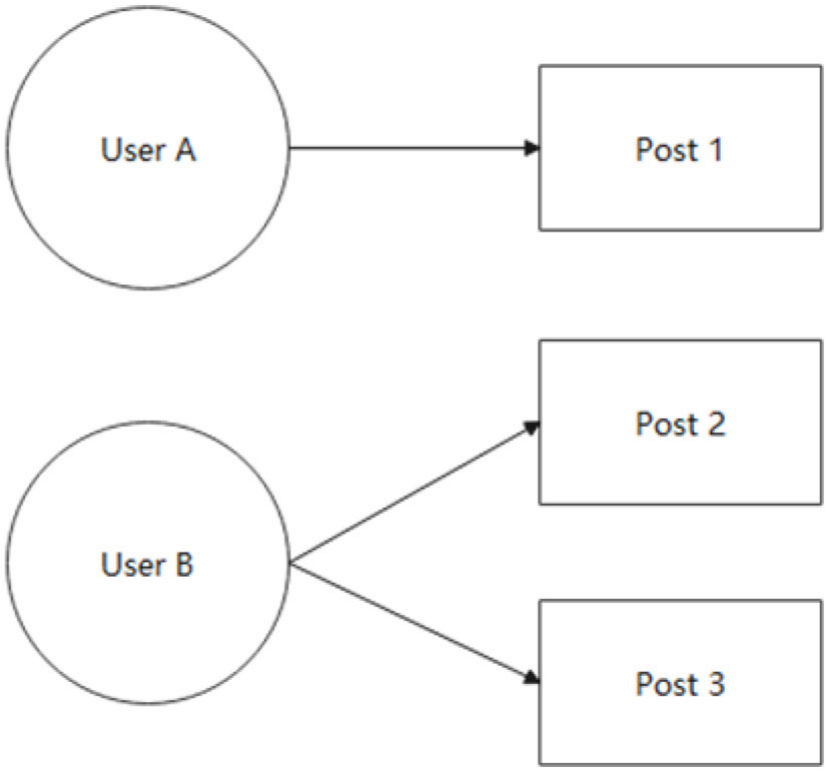
User posting diagram.

The research model is divided into sentiment analysis model and social network analysis model. In the sentiment analysis model, the dependent variable is the forward volume corresponding to each post in 3158 posts (*Forward*). In the social network analysis model, the same user may publish multiple different posts in real network relationships (Figure 4). According to the user name and user id, the sum of forwarding volume is processed, and the result is that 3158 posts are posted by 1440 different users, namely network node *g* = 1440. Therefore, the dependent variable in the social network analysis model is the sum of the retweets of each posting user of 1440 posting users (*Forward*_*sum*). If the user makes only one post, then *Forward* = *Forward*_*sum*; If the user makes multiple posts, then *Forward*>*Forward*_*sum*.

#### Independent variable

##### Sentiment analysis

In the sentiment analysis model, the relationship between positive sentiments and negative sentiments contained in the content of posts and the forwarding volume is mainly discussed. Therefore, the independent variable in the sentiment analysis model is the sentiment polarity score to which each post belongs. Since the sentiment analysis model adopts the method of grouping regression, the score of positive attributes in the independent variables is defined as (*positive*), and the score of negative attributes is defined as (*negative*). The score represents the emotional strength and is put in the regression model as an absolute value.

Weibo is a social platform mainly composed of short texts, and the text content is relatively short. Therefore, using the sentiment dictionary method to classify sentiments can achieve high accuracy. In this paper, using the DLUT sentiment vocabulary ontology database as an analysis tool, we get the values of independent variables in the sentiment analysis model. The principle of the sentiment dictionary method is to judge whether the text contains the sentiment words in the sentiment dictionary. If the content of this paper contains sentiment words, there is a sentiment tendency; if it does not contain any sentiment words, it is neutral. At present, the mainstream Chinese sentiment dictionaries include HowNet Sentiment Dictionary, NTSUSD Simplified Chinese Sentiment Dictionary, and Dalian University of Technology (DLUT) Sentiment Vocabulary Ontology Database (54). DLUT is a Chinese ontology resource sorted and annotated by the Information Retrieval Laboratory of Dalian University of Technology, which contains nearly 30,000 sentiment words. Its high coverage and accuracy of Chinese words have been widely used in sentiment analysis of Chinese texts (55). DLUT provides seven basic sentiments: good, happy, surprise, disgust, fear, anger, and sadness. It classifies the sentiment strength of words into five levels (1, 3, 5, 7, and 9). The higher the level, the stronger the sentiment is. At the same time, DLUT gives the specific sentiment score and sentiment polarity of each post according to the matching results of sentiment words. Posts with a sentiment score <0 are judged negative, and posts with a sentiment score > 0 are judged positive. Sentiment scores equal to 0 are judged as a neutral attribute. In the process of sentiment analysis, we wrote sentiment dictionary program based on Python (Anaconda3 5.3.0) and matched the research samples with DLUT sentiment vocabulary ontology database. Thus, the specific matching score of seven basic sentiments of each post is obtained, the corresponding score of sentiment polarity of the post is determined, and the independent variables in the sentiment analysis model of this paper are obtained.

##### Social network analysis model

In the social network analysis model, the influence of users' network relationship on forwarding behavior in the social network constructed in this paper is mainly discussed. On Weibo platform, users have already formed a huge social network through some connections. Therefore, this paper further investigates the influence of social network relation of posting users in the topic on its total forwarding volume and makes a regression analysis of degree, betweenness centrality, and closeness centrality of posting users as independent variables.

In the process of building the social network analysis model, we crawled the reposts of 3,158 posts, totaling 62,122 reposts. The main crawl information includes forwarding user id, forwarding user name, forwarding time, forwarding content, and forwarding posts id. On this basis, the posting users of 3158 posts were taken as network nodes, and the forwarding relations between 62,122 posting users and 3158 posting users were taken as edges to establish the initial “posting user-forwarding user” network. The network consists of two types of nodes, one is the posting user, and the other is the forwarding user, which is a two-mode network. It is worth clarifying that the network relationship in “posting user-forwarding user” established in this paper is an unweighted forwarding relations edge. That is, if the posting user has one or more forwarding relations in this network, they are regarded as having a forwarding relation, and the relationship between them is assigned a value of 1; if there are no forwarding relations between the posting user and the forwarding user, the relationship between them is assigned a value of 0. However, this paper focuses on the influence of the social network influence of posting users on the forwarding volume of posts. Since forwarding users may simultaneously forward the posts of multiple posting users, we regarded forwarding users as “Bridges” to establish connections between posting users and then transformed “posting user-forwarding user” network into “posting user-posting user” one model network. Gephi 0.9.5 software was used to construct the social network relationship of this paper, namely “Sina Weibo social network relationship of the topic of COVID-19 vaccines.” At the same time, according to Table 1, the values of degree, betweenness centrality, and closeness centrality of posting users in the network are calculated as independent variables in the social network analysis model.

**Table 1:** Method to measure the effectiveness of social network nodes.

**Type**	**Calculation formula**	**Measurement method description**
Degree	$degree_{i}=\frac{\underset{j}{\sum }X_{ji}}{g-1}$	*X*_*ji*_ represents the relationship between the posting user *i* and the posting user *j* through the reposting person, which is represented by a matrix association. If the posts of user *i* and user *j* are reposted by at least the same reposting person, the two establish a forwarding relationship, and *i* of the matrix The row *j* column is 1, otherwise it is 0. *g* represents the number of nodes in the network, that is, the number of posting users in the topic network of this research, (*g* = 1440), divided by (*g* − 1) for normalization.
Betweenness centrality	$betweenness_{i}=\frac{\underset{j<k}{\sum }g_{jk\left(ni\right)}/g_{jk}}{\left(g-1\right)\left(g-2\right)/2}$	*g*_*jk*(*ni*)_ represents the frequency of occurrence of posting user *i* in the shortest non-repeating path of posting user *j* and posting user *k*, and *g*_*jk*(*ni*)_/*g*_*jk*_ represents the degree of occurrence of posting user *i* in the shortest non-repeating path of forwarding relationship between other posting users. *g* is the number of nodes in the network, which is divided by (*g*−1)(*g*−2)/2.
Closeness centrality	$closeness={\left[\frac{\overset{g}{\underset{j=1}{\sum }}d\left(i,j\right)}{g-1}\right]}^{-1}$	*d*(*i, j*) represents the shortest distance between posting user *i* and posting user *j*. *g* is the number of nodes in the network, divided by (*g*−1).

#### Control variables

Existing studies show that posts with rich information form and content (e.g., external links, emoticons, and pictures) are more likely to attract public transmission, and the forwarding volume of these posts is significantly higher than that of posts with a single form (56). Therefore, the control variables in this paper include “Sum of posting volume of the user (*post*), the number of pictures contained in the post (*pic*), the total number of words in the post (*word*), the number of emojis (*emoji*) contained in the post, whether there will be external links (*url*), and the number of fans (*fans*) of the user.”

### Modeling and data analysis procedure

#### Empirical model

In this paper, we used multiple linear regression to establish grouping regression model (4-1, 4-2) for sentiment analysis and social network analysis model (4-3). Variable selection and measurement of model (4-1, 4-2, and 4-3) are shown in Table 2.


$$\begin{array}{l} Forward=\beta_{0}+\beta_{1}positive+\beta_{2\;}post+\beta_{3}word\\ +\beta_{4\;}pic+\beta_{5\;}emoji+\beta_{6\;}url+\varepsilon_{i}\;\left(4-1\right)\\ Forward=\beta_{0}+\beta_{1}negative+\beta_{2\;}post+\beta_{3}word\\ +\beta_{4\;}pic+\beta_{5\;}emoji+\beta_{6\;}url+\varepsilon_{i}\;\left(4-2\right)\\ Forward\text{\_}sum=\beta_{0}+\beta_{1}degree+\beta_{2\;}betweenness\\ +\beta_{3\;}closeness+\beta_{4\;}fans{+\varepsilon }_{i}\;\left(4-3\right) \end{array}$$

**Table 2:** Variables of sentiment analysis and social network analysis.

**Variable type**	**Variable symbol**	**Variable description**
**Dependent variable**	**Forward**	Forwarding volume
	**Forward_sum**	The sum of the retweets of each posting user of 1440 posting users
**Independent Variable**	**positive**	Positive attribute score
	**negative**	Negative attribute score
	**degree**	Social network structure degree centrality
	**betweenness**	Social network structure betweenness centrality
	**closeness**	Social network structure closeness centrality
**Control variables**	**post**	Sum of posting volume of the user
	**word**	Number of words of the post
	**pic**	Number of pictures
	**emoji**	Number of emojis contained in the post
	**url**	External links
	**fans**	Number of fans of the user
**Parameter**	**β_0_**	Constant term
	**β_*i*_(*i* = 1, 2, …6)**	Variable coefficient
	**ε_*i*_**	Error term

#### Data analysis procedure

In the process of data analysis, two sets of regression model data were analyzed and processed to ensure the interpretability of model results. First, to reduce the existence of extreme values or excessive differences between dependent variable (forwarding volume) data in the model, the forwarding volume in the model was logarithmically processed. To avoid the influence of extreme values on the results, statistical software Stata 17.0 was used to conduct 1% tail reduction. To make the regression coefficient of the negative group easy to understand, this paper takes the absolute value of the sentiment variable (negative score) in the regression of the negative group. To make the regression coefficients between variables more meaningful, Z-score standardization was used on all variables during group regression. In addition, considering the possible dependence between variables, bivariate correlation analysis was also included in the analysis process. Finally, the white heteroscedasticity robust standard error was used to adjust the *t*-value to avoid the impact of heteroscedasticity on the regression model results.

## Results

### Descriptive analysis

#### Ranking of retweets for “COVID-19 vaccine topic posts”

From the ranking of the retweets of Weibo posts on the topic of “COVID-19 vaccines” (Table 3), the top 50 posters had complex compositions, which included official media accounts (e.g., CCTV News, People's Daily, and Xinhua Net) and regional media accounts (e.g., Beijing Daily News and Shanghai Release). Individual users, including verified opinion leaders in the medical field such as Dr. Zhang Wenhong and Dr. Zeng Surgeon, as well as including uncertified individual users, who are also an important part of the Sina Weibo social network that constitutes the topic of COVID-19 vaccines. (Sina Weibo encourages identity verification, either as an individual or an organization. If the identity is verified, a symbol of “V” and the real identity are presented in the profile.).

**Table 3:** Ranking of retweets of the Weibo account “COVID-19 vaccine topic posts”.

**No**.	**User**	**Forward**	**No**.	**User**	**Forward**
1	CCTV News (OM)	11516	26	Metro Express (OM)	571
2	People's Daily (OM)	6384	27	CCTV.com (OM)	563
3	User 1 (IU)	4918	28	Geili Dujiangyan (RM)	538
4	User 2 (IU)	3869	29	Shanghai Life Podcast (RM)	530
5	Xinhua Net (OM)	2852	30	Chongqing Life Podcast (RM)	523
6	User 3 (IU)	2532	31	The paper (OM)	519
7	Xinhua News Agency (OM)	2396	32	Beijing Activity (RM)	502
8	Guoshizhitongche (OM)	2188	33	Beijing Daily News (RM)	501
9	Minnan Youth League Committee (RM)	2003	34	Sina News (OM)	479
10	China News Network (OM)	1755	35	Minhang Today (RM)	466
11	China Anti-Cult (OM)	1487	36	Observer Net (OM)	453
12	China Daily (OM)	1376	37	User 7 (IU)	433
13	Gulou Micro News (RM)	1073	38	Anhui Anti-cult (RM)	417
14	Life in Beijing (RM)	1007	39	State Grid Shaanxi Electric Power (RM)	409
15	Global Times (OM)	999	40	Weinan Observed (RM)	363
16	Things in Shenzhen (RM)	900	41	User 8 (IU)	360
17	Modern Express (OM)	863	42	Around Jinhua (RM)	325
18	People's Network (OM)	816	43	State Grid Hubei Electric Power (RM)	306
19	Guangzhou Daily (RM)	775	44	User 9 (IU)	306
20	User 4 (IU)	725	45	Guangxi Daily News (RM)	303
21	User 5 (IU)	683	46	Shanghai Release (RM)	283
22	Daily Economic News (OM)	676	47	China CDC (OM)	281
23	User 6 (IU)	627	48	Nominhe People's Procurator (RM)	270
24	The Communist Youth League of China (OM)	621	49	In Changsha (RM)	257
25	State Grid Jiangsu Electric Power (RM)	602	50	The Beijing News (RM)	256

^*^OM, represents official media accounts.

RM, represents regional media accounts.

IU, represents individual user.

#### Basic attributes of the “COVID-19 vaccine topic post” network

**Figure 5:**
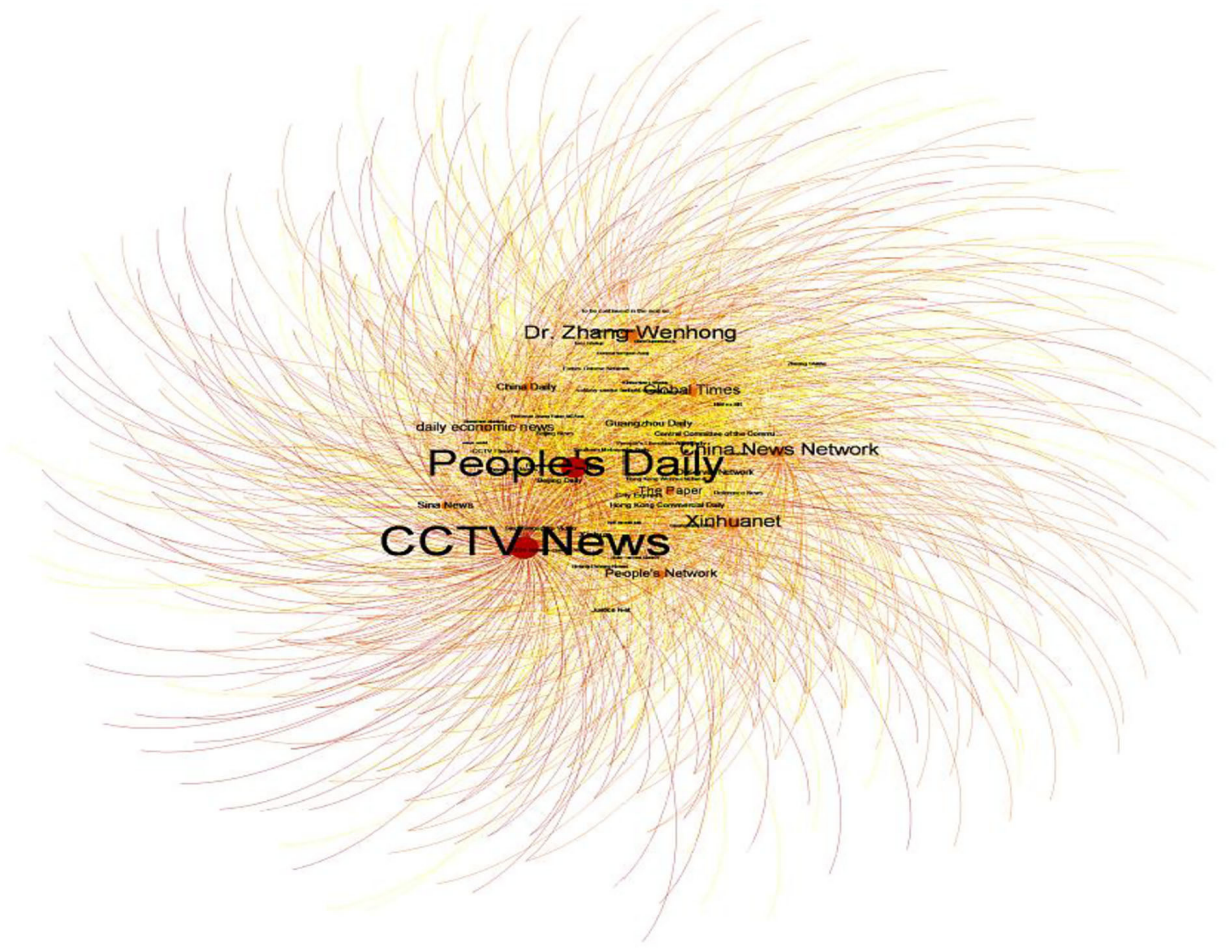
Sina Weibo social network diagram of COVID-19 vaccine topics.

See Figure 5 for the “COVID-19 Vaccine Topic on Sina Weibo Social network relationship” constructed in this paper. The basic properties of the “COVID-19 vaccine topic posts” network are obtained in Table 4. As indicated by the results, the 1440 nodes of the COVID-19 vaccine topic network established a total of 4993 connection pairs, with a social network density of 0.005, indicating that the network has 0.5% of network connections. It is a weakly dense network. This kind of weak dense network provides new health information and helps individuals access resources that are difficult to reach (57). The average distance between users posting was 2.786, indicating that any two posts relating to COVID-19 vaccines can arrive in less than three steps on average. Furthermore, the cohesion index of the network based on “distance” was 0.632, demonstrating that the web is a cohesive one.

**Table 4:** Basic attributes of the relationship network of COVID-19 vaccine topic posts.

**Attribute Name**	**Attribute Value**
Number of nodes	1440
Number of connections	4993
Density	0.005
Average distance	2.786
Diameter	8
Cohesion	0.632

#### Distribution of sentiment information in the sample of “COVID-19 vaccine topic posts”

According to the statistical results (Table 5), the Weibo posts on the topic of the COVID-19 vaccine topic all contain significant sentiments. Positive sentiment posts accounted for 54%, negative sentiment posts accounted for 25%, and neutral sentiment posts accounted for 21%. In addition, the number of positive sentiment posts was higher than the number of negative sentiment posts for both the top 50 forwarding users and the total sample. Accordingly, more positive sentiments than negative sentiments are conveyed in Weibo posts regarding the topic of the COVID-19 vaccines.

**Table 5:** Distribution of sentiment information in the sample.

	**Top 50 users who were retweeted (%)**	**Total sample size of posts (%)**
Positive	32 (64%)	1688(54%)
Neutrality	12(24%)	677 (21%)
Negative	6 (12%)	793 (25%)

### Regression analysis

#### Regression analysis of social network structure and sum of posts' forwarding

The statistical description of user characteristic variables, control variables, and forwarding volume in the social network analysis model is shown in Table 6. The results of bivariate correlation analysis among variables are shown in Table 7. The results of multiple linear regression and white heteroscedasticity robust standard error are shown in Table 8. The results show that the degree, betweenness centrality, and closeness centrality of posting users in the social network structure are significantly positively correlated with the forwarding volume of posts (β>0, *p* < 0.05). However, there was no significant regression between the number of fans and the forwarding volume (β = 0.224092, *p* = 0.39). The number of fans of posting users is positively correlated with the forwarding volume, which is consistent with

**Table 6:** Description of social network analysis indicators.

**Variable**	**Means**	**Standard deviation**
Forward_sum	38.83264	129.1646
Degree	6.184722	13.10202
Betweenness Centrality	0.0001959	0.0006657
Closeness Centrality	0.1012634	0.0987084
Fans	2336504	5675712

**Table 7:** Bivariate correlation analysis of social network analysis model.

	**Forward_sum**	**Degree**	**Betweenness Centrality**	**Closeness Centrality**	**Fans**
Forward_sum		0.678^***^	0.625^***^	0.572^***^	0.369^***^
Degree	0.622^***^		0.767^***^	0.905^***^	0.390^***^
Betweenness Centrality	0.540^***^	0.835^***^		0.727^***^	0.400^***^
Closeness Centrality	0.532^***^	0.544^***^	0.372^***^		0.429^***^
Fans	0.410^***^	0.643^***^	0.527^***^	0.344^***^	

Lower-triangular cells report Pearson's correlation coefficients, and upper-triangular cells are Spearman's rank correlation.

*** p <0.01, ** p <0.05, and * p <0.1.

**Table 8:** Multiple linear regression results of social network structure.

**Forward_sum**	**Coefficient**	**Std. err**.	**T**	**P**	**[95% conf**.	**interval]**	**VIF**	**1/VIF**
Degree	0.3251829	0.0426274	7.63	0	0.2415641	0.4088017	4.69	0.201639
Betweenness Centrality	0.1477294	0.0343723	4.3	0	0.0803042	0.2151547	3.41	0.292869
Closeness Centrality	0.2926066	0.024674	11.86	0	0.2442056	0.3410077	1.71	0.586327
Fans	0.0224092	0.0260823	0.86	0.39	−0.0287544	0.0735727	1.47	0.681897
**R–squared:**	**0.4469**							

our common cognition, but the coefficient is not significant. This indicates that under the shadow of social network relationship, the influence of users' social network on the forwarding volume is significantly stronger than the number of fans.

#### Regression analysis of textual sentiment and sum of posts' direct forwarding

The statistical description of sentiment variables, control variables, and forwarding volume in the sentiment analysis model is shown in Table 9. Tables 10, 11 show the results of bivariate correlation analysis among variables. The results of multiple linear grouping regression and white heteroscedasticity robust standard error are shown in Tables 12, 13. The results show that there is no significant correlation between posts containing more positive sentiments and the forwarding volume (β = 0.0307029, *p* = 0.115), and there is a significant positive correlation between posts containing more negative sentiments and the forwarding volume (β = 0.0702364, *p* = 0.013). This indicates that posts containing negative sentiments are more likely to be forwarded, and it is worth paying more attention to the specific reasons.

**Table 9:** Description of sentiment analysis indicators.

**Variable**	**Means**	**Standard deviation**
Forward	16.3695	49.4436
Positive score	9.742	8.3283
Negative score	−5.6617	3.7648
Posts	55,918.174	52,043.7016
Pic	0.7276	1.8967
Emoji	0.1450	0.4718
link (ref. = no link)	0.5675	0.4957

**Table 10:** Bivariate correlation analysis of positive sentiment regression model.

	**Forward**	**Positive**	**Posts**	**Pic**	**Word**	**Emoji**	**ink (ref. = no link)**
**Forward**		**0.021**	0.199^***^	**−0.004**	**0.022**	**−0.015**	**−0.029**
**Positive**	**0.037**		**−0.025**	0.090^***^	0.252^***^	**0.039**	−0.081^***^
**Posts**	0.181^***^	−0.041^*^		−0.045^*^	0.102^***^	**−0.036**	0.054^**^
**Pic**	**0.016**	0.055^**^	−0.065^***^		0.178^***^	**−0.032**	−0.455^***^
**Word**	**0.021**	0.440^***^	**0.037**	0.078^***^		−0.178^***^	−0.144^***^
**Emoji**	**−0.008**	**0.019**	−0.065^***^	0.047^*^	−0.114^***^		0.115^***^
**ink (ref. = no link)**	**−0.017**	−0.113^***^	**0.015**	−0.307^***^	−0.229^***^	0.093^***^	

Lower–triangular cells report Pearson's correlation coefficients, and upper–triangular cells are Spearman's rank correlation.

*** p <0.01,

** p <0.05, and

* p <0.1.

**Table 11:** Bivariate correlation analysis of negative sentiment regression model.

	**Forward**	**Negative**	**Posts**	**Pic**	**Word**	**Emoji**	**ink (ref. = no link)**
Forward		0.181^***^	0.246^***^	−0.025	0.118^***^	−0.061^*^	0.014
Negative	0.150^***^		0.080^**^	0.042	0.243^***^	−0.028	−0.056
Posts	0.217^***^	0.070^**^		−0.107^***^	0.124^***^	−0.111^***^	0.204^***^
Pic	0.001	0.022	−0.066^*^		0.060^*^	0.052	−0.385^***^
Word	0.091^***^	0.359^***^	−0.023	0.027		−0.213^***^	−0.008
Emoji	−0.059^*^	−0.049	−0.127^***^	0.079^**^	−0.132^***^		0.071^**^
ink (ref. = no link)	0.012	−0.067^*^	0.166^***^	−0.256^***^	−0.134^***^	0.069*	

Lower–triangular cells report Pearson's correlation coefficients, and upper–triangular cells are Spearman's rank correlation.

*** p <0.01,

** p <0.05, and

* p <0.1.

**Table 12:** Multiple linear regression results of positive sentiment regression model.

**Forward**	**Coefficient**	**Std. err**.	**T**	**P**	**[95% conf**.	**interval]**	**VIF**	**1/VIF**
Positive	0.0307029	0.019487	1.58	0.115	−0.00752	0.068924	1.25	0.797561
Posts	0.05303	0.020627	2.57	0.01	0.012572	0.093488	1.5	0.664573
Pictures	0.0321524	0.020503	1.57	0.117	−0.00806	0.072367	1.12	0.895237
Word	−0.0007483	0.021699	−0.03	0.972	−0.04331	0.041811	1.32	0.756767
Emoji	0.004695	0.021157	0.22	0.824	−0.0368	0.046191	1.04	0.965141
link (ref. = no lin)	0.0087473	0.019094	0.46	0.647	−0.0287	0.046199	1.17	0.853347
**R–squared**	**0.4229**							

**Table 13:** Multiple linear regression results of negative sentiment regression model.

**Forward**	**Coefficient**	**Std. err**.	**T**	**P**	**[95% conf**.	**interval]**	**VIF**	**1/VIF**
Negative	0.0702364	0.0283035	2.48	0.013	0.0146764	0.1257963	1.17	0.854668
Posts	0.085584	0.0316312	2.71	0.007	0.023492	0.1476761	1.46	0.683312
Pictures	0.0482189	0.0336267	1.43	0.152	−0.0177905	0.1142282	1.09	0.92077
Word	0.0553122	0.0321203	1.72	0.085	−0.00774	0.1183644	1.19	0.840618
Emoji	−0.0213912	0.030262	−0.71	0.48	−0.0807956	0.0380132	1.05	0.948691
link(ref. = no lin)	0.015699	0.0301194	0.52	0.602	−0.0434254	0.0748235	1.14	0.876393
**R–squared:**	**0.3725**							

## Discussion

### The effect of posting user characteristics on the post forwarding

The first result of this paper was that the higher the degree of posting users in the social network structure, the higher the forwarding volume. This research result is consistent with previous research conclusions (36). It indicates that the research topic does not affect the difference of research results, and users with high degrees are more likely to have their posts forwarded. The object of the previous study was a vaccine issue, but the object of this paper was the COVID-19 vaccine, which did not affect the influence of opinion leaders in the process of reposting. In the 1940s, Lazarsfeld put forward the concept of opinion leader. The research finds that there are two levels of transmission in information transmission. Mass transmission does not directly spread ideas to the general audience but needs to pass through the intermediate link of opinion leaders and then convey them to the less active public. The results of this paper confirm that opinion leaders still retain the ability to influence audiences in the specific vaccine topic network structure of COVID-19, and their influence can lead to a large forwarding volume of COVID-19 vaccine posts. Therefore, attention should be paid to the importance of opinion leaders in the process of vaccine information transmission.

The second result of this paper was that the betweenness centrality of post users was positively correlated with the forwarding volume of posts, consistent with previous findings. As revealed by the findings, posts posted by users with gatekeeper characters are more likely to be reposted. For the traditional media, editors often play the gatekeeper role. However, in social media, the role of gatekeeper is more diversified since some ordinary users can also act as gatekeepers. Users are consumers of information and gatekeepers of information (58). It is noteworthy that according to the statistics of the top 50 betweenness centrality in the network structure of “COVID-19 vaccine topics,” there were many ordinary users' accounts (see Supplementary material). It is because ordinary users act as gatekeepers in disseminating COVID-19 vaccine information that the amount of COVID-19 vaccine information has increased geometrically. In the process of information gatekeeping for COVID-19 vaccine information, the efficiency of information gatekeeping by official media is relatively low. Meantime, ordinary users can immediately post the information on the Weibo platform *via* self-gating, followed by other users seeing the information to decide whether to forward it or not, which results in a rapid spread of COVID-19 vaccine information. This paper confirmed the vital role of “gatekeeper” users in disseminating COVID-19 vaccine information and found the possible meaningful effect of ordinary users' participation in the gatekeeping of COVID-19 vaccine information.

The third result of this paper was that the closeness centrality of users was positively correlated with the forwarding volume of posts. In the previous literature review section, we reviewed users' closeness centrality. Previous literature only shows the relationship between closeness centrality and user access to information. Users with high-closeness centrality are relatively independent in the actual complex network structure of information transmission and can obtain useful information through a small number of nodes. Our results further expand the relationship between closeness centrality and forwarding volume. Therefore, we speculate that users with high-closeness centrality have an advantage not only in obtaining useful information, but also in disseminating information. In particular, in the particular social network structure of COVID-19 vaccine, high-closeness centrality users are not only receivers of information, but also important transmitters of information. In other words, the transmission of COVID-19 vaccine information needs to focus on the influence of high-closeness central users.

### Sentiment and the effect of COVID-19 vaccine information transmission

This paper aimed to understand the correlation between different sentiment posts and the sum of posts' direct forwarding volume. This paper showed a positive correlation between negative sentiment posts and sum of posts' direct forwarding volume, while positive sentiment posts were not significantly correlated with sum of posts' direct forwarding volume. This finding extends the correlation between post sentiment and forwarding behavior. It gives us an insight into the correlation between sentiment and forwarding behavior, specifically which is within the COVID-19 vaccine topic. Combined with Table 4: the distribution of sentiment information in the sample, posts containing negative sentiments accounted for nearly 25% of the total sample, indicating that negative sentiments cannot be ignored in the discussion of COVID-19 vaccine and may have formed a certain influence.

To be specific, we found posts could be divided into two main types in terms of post content. A post may positively impact vaccination, whereas the other type has a negative effect. The first type refers to the refute rumor posts published by official media accounts and the popularization posts of the knowledge of COVID-19 vaccines by official media accounts. For instance, the Weibo user “CCTV News” posted a post with the tag “Who shouldn't be vaccinated with COVID-19 vaccines,” the content of which provides specific information regarding the people who are not suitable to be vaccinated with the COVID-19 vaccines. The Weibo user, “People's Daily,” posted a post with the tag “10 questions and answers to gain insights into the newest doubts regarding vaccination,” covering various aspects of COVID-19 vaccines vaccination. The Weibo user “Guangzhou Daily” posted a message with the hashtag “False news concerned with the first vaccination stoppage in Guangzhou after June 9” to refute rumors. Although the posts mentioned above were defined as negative posts for containing more negative sentiment words, the actual impact was positive. Another type of Weibo post, mostly by ordinary users and primarily related to the vaccine's side effects, was more negative and had a high number of forwarding volumes. For instance, the Weibo user ordinary users A (3869 forwarding volume) said in a post, “Today I was given a preliminary diagnosis of sudden deafness. I think it's a side effect of the vaccine. My diet, lifestyle, etc., is the same as before the vaccine, which is why I suspect it's the vaccine. Online also a lot of people said that after the vaccine tinnitus ear stuffy hearing decline.” A post tagged “COVID-19 vaccines side effects” was posted by Weibo user ordinary users B (forwarding volume: 2532), and the text content is, “I don't know if it has anything to do with the COVID-19 vaccine, but I developed symptoms nine days after I finished the vaccine. There was nothing wrong with my ears before.” Although posts like the mentioned reflect the true feelings of individuals after receiving the COVID-19 vaccines, whether the COVID-19 vaccines directly cause harm (e.g., deafness without scientific confirmation from medical institutions) remains unclear. It has been studied that knowing that a person has experienced the side effects of influenza vaccination is a valid cue to action that affects vaccination behavior and can harm the influenza vaccination program (59). Therefore, the spread of such information is also likely to hinder the vaccination and popularization of the COVID-19 vaccines.

## Conclusion and enlightenment

Based on social media data, this paper examines the impacts of Weibo text sentiment and users' social network characteristics on post forwarding in the COVID-19 vaccine topic. The results show that posts relating to COVID-19 vaccines with negative sentiment and neutral sentiment are more likely to be forwarded on social media, and posts relating to COVID-19 vaccines by posters with higher betweenness centrality or lower degree are more likely to be forwarded on social media. In view of the above conclusion, this paper puts forward the following recommendations.

The first suggestion of this paper is to emphasize opinion leaders, gatekeeper users, and high-closeness centrality users. The research results of this paper show that users with high degrees, betweenness centrality, and closeness centrality are more likely to have their posts forwarded. Therefore, accounts with such characteristics in government, media, and medical institutions should give full play to their advantages in the transmission of COVID-19 vaccine information. On the basis of authentic and scientific information, the number of posts on COVID-19 vaccine knowledge will be increased to effectively improve the spread of COVID-19 vaccine posts. In addition, previous studies have shown that if accounts with high betweenness centrality do not follow a certain information, it is difficult for the information to flow to another transmission field (34). Therefore, “gatekeeper” users with high betweenness centrality need to fully understand the public's demand for COVID-19 vaccine information and select appropriate information to publish on social media platforms to effectively convey to the audience.

The second recommendation is to mitigate the negative effects of the information epidemic. This paper found that a certain percentage of those users having high betweenness centrality were ordinary users (see Supplementary material). This suggests that the participation of ordinary users in the gate-keeping process of COVID-19 vaccine information on social media platforms, especially in the social network structure of the COVID-19 vaccine topic, has led to a geometric increase in the flow of information. Although ordinary users' participation in the gate-keeping process of COVID-19 vaccine information is less time-consuming and more efficient than official media accounts because they merely “self-gate” the posts, it may negatively impact an event information epidemic. Accordingly, the relevant departments should facilitate the monitoring of information and draw upon technological tools [e.g., big data (60) technology and artificial intelligence (61)] to improve the prevention and management of information epidemics to reduce the negative effect of information epidemics.

The third recommendation is to enhance knowledge of the side effects of the COVID-19 vaccines. As indicated by this paper, more negative posts were positively correlated with the forwarding volume of posts, and the further discussion revealed that the implications of this finding are 2-fold. Though the government, media, and medical institutions have posted on social media about the COVID-19 vaccines and refuted the rumors regarding the COVID-19 vaccines, some posts concerned with the vaccine's side effects were still reposted on social media. Hence, it is necessary to think deeply about the reasons behind this. First, we analyzed the popularization of knowledge about COVID-19 vaccines and found that the content about the adverse reactions to the COVID-19 vaccines accounted for relatively little. Second, when the public experienced adverse reactions, there was a lack of scientific proof from relevant institutions that vaccination was the cause of the adverse reactions. Thus, first, the government needs to strengthen the popularization of knowledge about the side effects of the COVID-19 vaccines. Next, the services offered by the government and medical institutions should be improved in response to adverse reactions in public after vaccination, and the causes of adverse responses should be scientifically determined to mitigate the negative effect of posts about the side effects of the COVID-19 vaccines on social media.

## Limitations and future research

Despite this paper analyzing the correlation between social network characteristics, textual sentiment, and forwarding of posts relating to COVID-19 vaccines, however, there are some limitations: First, due to time constraints, although this paper obtained a lot of original data, after data cleaning, there were relatively few data in line with the research topic, so future studies can supplement more data. Second, the mechanism of other factors impacting the forwarding of posts relating to COVID-19 vaccines, besides social network characteristics and textual sentiment, still needs to be further explored. Third, because this paper only studied China's Weibo users, most of them were young adults, with children and elderly groups seldom involved. For this reason, this paper is of limited reference significance for other groups. Lastly, this paper only explored the transmission effect of the COVID-19 vaccine topic from the perspective of transmission scope. However, the actual transmission effect of the COVID-19 vaccines message still needs to be further investigated due to the information epidemic influences it. Accordingly, subsequent studies can more comprehensively discuss and analyze the measurement of relevant variables and the reception of COVID-19 vaccine information among the elderly and children's groups. In addition, further studies on the actual effects of COVID-19 vaccine information transmission can be conducted in future.

## Data availability statement

The raw data supporting the conclusions of this article will be made available by the authors, without undue reservation.

## Ethics statement

The studies involving human participants were reviewed and approved by Institutional Review Board of Social Sciences and Humanities Training Program, Jinan University. Written informed consent for participation was not required for this study in accordance with the national legislation and the institutional requirements.

## Author contributions

KS designed the study and analyzed the data. HW and KS were involved in manuscript writing. JZ revised the manuscript. All authors have read and approved the manuscript.

## Funding

This paper was funded by the National Social Science Foundation of China (Grant No. 15ZDA042).

## Conflict of interest

The authors declare that the research was conducted in the absence of any commercial or financial relationships that could be construed as a potential conflict of interest.

## Publisher's note

All claims expressed in this article are solely those of the authors and do not necessarily represent those of their affiliated organizations, or those of the publisher, the editors and the reviewers. Any product that may be evaluated in this article, or claim that may be made by its manufacturer, is not guaranteed or endorsed by the publisher.
