# Supplementary material for: The impact factors of social media users' forwarding behavior of COVID-19 vaccine topic: Based on empirical analysis of Chinese Weibo users
Source: Front Public Health. 2022 Sep 14;10:871722. doi: 10.3389/fpubh.2022.871722 (PMC9515960; doi:10.3389/fpubh.2022.871722)
Supplement: Supplementary file 1 [file Data_Sheet_1.PDF]

Supplementary material

Table 1: Classification results of posting content and research subject-related content

No.	User	Posting time	Forwarding volume	Post content	Content classification results
1	User A	2021-01-07 22:03	1847	I would like to ask netizens whether there are any side effects after taking the COVID-19 vaccine? Or an adverse reaction? Our school is due to collect the list at 12 noon tomorrow, but I am hesitant to get vaccinated. Because I'm afraid of pain and side effects. Are there any netizens who have been vaccinated? Shall we discuss it? Thank you.	1= Relevant
2	User B	2021-02-21 21:10	1660	Can people with allergic rhinitis be vaccinated against COVID-19? Among the contraindications in the COVID-19 vaccine instructions are patients who have had a severe allergic reaction to vaccination in the past (such as acute anaphylaxis, urticaria, skin eczema, dyspnea, angioneurotic edema, or abdominal pain) should not be vaccinated. So, is allergic rhinitis within the scope of inoculation? I would like to know netizens who have similar experiences, what side effects did you have after being vaccinated? Or is there anything to pay attention to?	1= Relevant
3	User C	2021-04-27 19:19	2277	I went to get a single shot of the COVID-19 vaccine yesterday, and after 16.30 in the afternoon, I felt nothing. I read a lot of posts about the side effects of vaccination before getting the vaccine, however, I didn't have much adverse reactions after the actual vaccination. So, I am guessing, is the adverse reaction related to the type of vaccine vaccinated? Friends, what kind of vaccine are you all vaccinated against? Is it Sinovac COVID-19 Vaccine? Or CanSino BIO vector vaccine?	1= Relevant
4	User D	2021-05-13 22:18	3869	# Side effects of COVID-19 vaccine. #COVID - 19 # #attention# sudden deafness<br>Today I was given a preliminary diagnosis of sudden deafness. I think it's a side effect of the vaccine. I was vaccinated on the morning of 7th. I feel stuffy in my ears on the morning of the 12th and it continues to be stuffy. My diet, lifestyle, etc., is the same as before the vaccine, which is why I suspect it's the vaccine. Online also a lot of people	1= Relevant

				said that after the vaccine tinnitus ear stuffy hearing decline. If you have the same reaction, you should report it to the vaccination site and the local CDC.	
5	User D	2021-05-21 21:58	2532	# Side effects of COVID-19 vaccine. I have lost the hearing in my right ear for five days. On the evening of the 16th, I suddenly felt a loud ringing in my ears. In the morning of the 17th, I couldn't hear anything in my right ear. I went to two hospitals and was diagnosed with sudden nerve deafness. The doctor says I need to be hospitalized, and it's doubtful I'll ever be cured. (I don't know if it has anything to do with the COVID-19 vaccine, but I developed symptoms nine days after I finished the vaccine. There was nothing wrong with my ears before.	1= Relevant

Table 2: Classification results of irrelevant posting content and research topic

No.	User	Posting time	Forwarding volume	Post content	Content relevance
1	User E	2021-01-01 20:30	170	about 7 billion doses of COVID-19 vaccine have been subscribed globally]<br>Some 7 billion doses of COVID-19 vaccine have been ordered globally as of December 18, according to data released by Duke University in the US. This includes more than 4 billion doses in developed countries, more than 1.1 billion doses in upper-middle-income countries and more than 1.8 billion doses in lower-middle-income countries. In addition, low-income countries subscribe to zero vaccines.	0= irrelevant
2	User F	2021-01-03 22:57	40	[18 enterprises have started production capacity construction of COVID-19 vaccine]<br>In order to accelerate the industrialization process of COVID-19 vaccine, the Ministry of Industry and Information Technology has set up a special team on production and guarantee of COVID-19 vaccine. Weekly scheduling of key enterprises capacity construction schedule.	0= irrelevant
3	User G	2021-01-06 21:39	1514	Gold NEWS: The number of COVID-19 cases continues to rise, lifted by a weaker DOLLAR and growing investor concerns over the pandemic. The slower-than-expected pace of	0= irrelevant

				COVID-19 vaccination has also sustained gold's gains. "Given the increased risk associated with the virus, in the short term we see people moving money to safe havens," said TIAA Bank's president of global markets.	
4	User H	2021-01-20 21:44	215	NBA Commissioner Silver said the league is in discussions to COVID-19 vaccination NBA players against the coronavirus, US media reported.	0= irrelevant
5	User I	2021-03-04 19:53	321	China's first batch of COVID-19 vaccines has arrived in Iraq, a timely arrival. Just hours after its arrival, Iraq's Ministry of Health immediately launched a national vaccination program.	0= irrelevant

Table 3: The top 50 users of betweenness centrality

No.	User	Forwarding volume	Betweenness centrality
1	CCTV News	11516	0.063782
2	People's Daily	6384	0.056414
3	User 1 (Individual user)	4918	0.029953
4	User 2 (Individual user)	1660	0.025132
5	Xinhuanet	2852	0.024857
6	Global Times	999	0.014552
7	The Paper	519	0.01083
8	People's Network	816	0.008673
9	daily economic news	676	0.008185
10	Sina News	479	0.007994
11	China Daily	1376	0.007083
12	Xinhua News Agency	2396	0.006892
13	Guangzhou Daily	775	0.006013
14	Observer Network	453	0.004768
15	Hong Kong Commercial Daily	128	0.004696
16	City Express	571	0.004376
17	The Communist Youth League of China	621	0.004321
18	TouTiao News	226	0.003735
19	Beijing Daily	200	0.003651
20	CCTV Finance	158	0.003618
21	Beijing News	256	0.003516
22	China News Videos	172	0.003432
23	Sina Finance	96	0.002932
24	Reference News	161	0.002809
25	User 3 (Individual user)	3869	0.002772
26	Justice Net	85	0.002747

27	Financial Network	150	0.00274
28	User 4 (Individual user)	1847	0.002618
29	User 5 (Individual user)	2277	0.002618
30	User 6 (Individual user)	2532	0.002582
31	Southern Metropolis Daily	83	0.002564
32	Sina Military	149	0.00256
33	CCTV.com	563	0.002328
34	China News Weekly	42	0.002248
35	CGTN	228	0.002235
36	Hong Kong Wenhui Network	92	0.002234
37	People's Liberation Army Daily	156	0.002208
38	Beijing Evening News	178	0.002203
38	Forbes Chinese Network	100	0.002185
39	Guangming Daily	23	0.00218
40	Lychee News	167	0.002164
41	User 7 (Individual user)	104	0.001925
42	User 8 (Individual user)	433	0.001825
43	Micro world	73	0.001807
44	User G (Individual user)	252	0.001786
45	Guangdong Communist Youth League	50	0.001786
46	Sina News Client	53	0.001718
47	People's Daily Overseas Edition - Overseas Network	61	0.001656
48	boss hookup	66	0.001631
49	User 9 (Individual user)	63	0.001618
50	User 10 (Individual user)	41	0.001614
